# Rapid emergence of ESBL producers in *E. coli* causing urinary and wound infections in Pakistan

**DOI:** 10.12669/pjms.292.3144

**Published:** 2013-04

**Authors:** Muhammad Asif Habeeb, Yasra Sarwar, Aamir Ali, Muhammad Salman, Abdul Haque

**Affiliations:** 1 Muhammad Asif Habeeb, Health Biotechnology Division, National Institute for Biotechnology and Genetic Engineering (NIBGE), P.O.Box 577, Jhang Road, Faisalabad, Pakistan, An affiliated Institute of Pakistan Institute of Engineering and Applied Sciences, Nilore, Islamabad, Pakistan.; 2 Yasra Sarwar, Health Biotechnology Division, National Institute for Biotechnology and Genetic Engineering (NIBGE), P.O.Box 577, Jhang Road, Faisalabad, Pakistan, An affiliated Institute of Pakistan Institute of Engineering and Applied Sciences, Nilore, Islamabad, Pakistan.; 3 Aamir Ali, Health Biotechnology Division, National Institute for Biotechnology and Genetic Engineering (NIBGE), P.O.Box 577, Jhang Road, Faisalabad, Pakistan, An affiliated Institute of Pakistan Institute of Engineering and Applied Sciences, Nilore, Islamabad, Pakistan.; 4 Muhammad Salman, Health Biotechnology Division, National Institute for Biotechnology and Genetic Engineering (NIBGE), P.O.Box 577, Jhang Road, Faisalabad, Pakistan, An affiliated Institute of Pakistan Institute of Engineering and Applied Sciences, Nilore, Islamabad, Pakistan.; 5 Abdul Haque, Health Biotechnology Division, National Institute for Biotechnology and Genetic Engineering (NIBGE), P.O.Box 577, Jhang Road, Faisalabad, Pakistan, An affiliated Institute of Pakistan Institute of Engineering and Applied Sciences, Nilore, Islamabad, Pakistan.

**Keywords:** Pathogenic *E. coli*, ESBL production, Drug resistance, Genotypic characterization

## Abstract

***Objectives:*** Production of extended spectrum beta -lactamases (ESBLs) by clinical isolates of pathogenic *E. coli* is a very serious therapeutic threat. This study was aimed to investigate the prevalence of ESBLs and associated drug resistance in *E. coli* isolates from urine and pus, and to report the drift from 2005 to 2009-10.

***Methodology:*** Among 173 *E. coli *isolates, 82 were phenotypically detected as ESBL producers by standard cefotaxime / clavulanic acid and ceftazidime / clavulanic acid disc diffusion tests. Antimicrobial resistance of all ESBL producers was assessed by disc diffusion method. Presence of CTX-M, TEM, SHV and OXA groups was investigated by PCR.

***Results:*** The prevalence of ESBL producing *E. coli* increased significantly from 33.7% in 2005 to 60.0% in 2009-10 (urine: 31.8% to 62.9%; pus: 41.1% to 55.5%). Resistance to cefotaxime, ceftazidime, ciprofloxacin, gentamicin, nalidixic acid, ticarcillin-clavulanic acid, and trimethoprim-sulfamethoxazole was above 85% in both sets of isolates. Imipenem and Fosfomycin resistance was non-existent in 2005 but ranged from 3-15% in 2009-10. Remarkable increase from 9.5% to 64.7% in urinary tract isolates and from 0 to 55% in pus isolates was observed in colistin sulphate resistance. The dissemination of genes encoding ESBLs was: CTX-M 3.5%; TEM 10.7%; both CTX-M and TEM 3.5% in 2005, and CTX-M 42.5%; TEM 48.1%; both CTX-M and TEM 29.6% in 2009-10.

***Conclusions:*** Our results showed very rapid emergence of multidrug resistant ESBL producing *E. coli* in Pakistan posing a very serious threat in the treatment of nosocomial and community acquired infections.

## INTRODUCTION

*Escherichia coli* (*E. coli*) have transcended to a level of antimicrobial resistance where humans are fronting severe clinical challenges in the form of urinary tract infections (UTIs), surgical wounds infections and neonatal sepsis.^1^^,^^2^ *E. coli *are presenting quick spread of resistance genes between strains by horizontal transfer mainly by plasmids.^2^ Production of beta -lactamases is the major defense tool of Enterobacteriaceae against β lactam antibiotics. These bacteria are capable of reducing efficacy of modern extended spectrum cephalosporins except cephamycins and carbapenems by the production of plasmid mediated extended spectrum beta -lactamases (ESBLs). The main genes involved in ESBL production include TEM genes (*bla*_TEM_), SHV genes (*bla*_SHV_), OXA genes (*bla*_OXA_) and CTX-M genes (*bla*_CTX-M_). Variants of TEM and SHV type ESBLs were exclusively prevalent until late 1990s but currently ESBLs are varying this characteristics dramatically with CTX-M producers replacing TEM and SHV mutants in European countries and other parts of the world using *E. coli* as major host.^3^ CTX-M producing *E. coli *causing urinary tract infections are associated with co-production of various β-lactamases e.g. TEM, and other resistance tools such as aminoglycoside modifying enzymes.^4^ High level dissemination of ESBL producing *E. coli* in nosocomial and community acquired infections is stated in most of the regions of world.^5^ These organisms are non-susceptible to most of the antimicrobials used for the treatment of UTIs and wound infections such as ciprofloxacin, gentamicin, trimethoprim-sulfamethoxazole and most of the cephelosporins.^6^ Very limited data are available on the prevalence of ESBLs in Pakistan and very few studies are published on genetic characterization of these enzymes. One recent study found the prevalence of CTX-M, TEM and SHV as 59%, 21% and 16% respectively in pathogenic *E. coli* isolates from a tertiary care health facility in Pakistan.^7^

The aim of this study was to investigate the prevalence of ESBL producing *E. coli* pathogens isolated from urine and pus samples collected from different geographical regions of Pakistan in two different time periods, 2005 and 2009-10. The isolates were characterized by molecular techniques and comparison was done to find the drift in numbers and characteristics in 5 years.

## METHODOLOGY

***Bacterial Isolates: ***The first batch was constituted by 83 *E. coli* isolates (66 from urine and 17 from pus samples) collected in 2005 from different hospitals of Pakistan, characterized and kept in NIBGE stock cultures. The second batch comprised of 90 *E. coli* isolates (54 from urine and 36 from pus samples) collected during 2009-10.

All the isolates were revived in trypticase soy broth (TSB) (Merck, Darmstadt, Germany), sub-cultured on MacConkey agar (Merck, Darmstadt, Germany) to get isolated colonies, and identified by conventional biochemical methods and commercially available kits (API 20E bioMérieux Marcy I’Etoile, France and rapID Systems, Remel, Lenexa, Kansas, USA).

***Detection of ESBL Phenotypes: ***Mueller Hinton agar (Merck, Darmstadt, Germany) was used for initial screening and phenotypic confirmation of ESBL producing *E. coli* isolates using CLSI guidelines for disc diffusion methods.^8^ Antimicrobial discs used were ceftazidime alone and with clavulanic acid; and cefotaxime alone and with clavulanic acid. An isolate showing enhanced zone of ≥5mm with antibiotic combined with clavulanic acid as compared to a zone of antibiotic alone on a disc was phenotypically designated as ESBL producer.

***Antimicrobial Susceptibility Testing: ***All phenotypically positive ESBL producing *E. coli *were further investigated for detection of antimicrobial resistance by disc diffusion methods using CLSI guidelines.^8^ Isolates were tested against conventional antimicrobials cefotaxime, ceftazidime (cephalosporins), nalidixic acid (quinolones), ciprofloxacin (flouroquinolones), gentamicin (aminoglycosides), ticarcillin-clavulanic acid (augmented penicillins), and trimethoprim-sulfamethoxazole (sulphonamides); and antimicrobials used in treatment of ESBL producing *E. coli*: imipenem (carbapenems), fosfomycin (phosphonic acids) and colistin sulphate (polymyxins).

***Molecular Detection of ESBLs: ***Total genomic DNA was extracted by phenol-chloroform method^9^ from all ESBL producing *E. coli* isolates using overnight growth in TSB. The beta -lactamase genes (*bla*_CTX-M_*, bla*_TEM_*, bla*_SHV_* and bla*_OXA_) were targeted using forward and reverse primers listed in Table-I.

All PCR assays (with 50 µl of each reaction mixture) were carried out in a thermal cycler (ATC201 NYX Technik, USA) with following reagents in addition to template: 10X buffer 5µl, 2.5mM of MgCl_2_, 50µM of each dNTP, 1.5U of *Taq *polymerase (Fermantas, Maryland, USA), 0.3µM of each primer (Gene Link, Hawthorne, NY, USA) and deionized water to make the volume.

Thermal cycler conditions for CTX-M gene were: initial denaturation at 94^o^C for 7 minutes, 35 repeats of denaturation at 94^o^C for 60 sec, annealing at 55^o^C for 40 sec and extension at 72^o^C for 60 sec while final extension was conducted at 72^o^C for 5 minutes. For TEM gene, the conditions were: 96^o^C for 5 minutes, 35 repeats of 96^o^C for 60 sec, 58^o^C for 60 sec and 72^o^C for 60 sec while final extension was conducted at 72^o^C for 10 minutes. Thermal cycler conditions for SHV and OXA were same as for TEM PCR; the only difference was in annealing temperatures (65^o^C and 60^o^C for SHV and OXA respectively). PCR products were separated on 1.5% agarose gel (Vivantis, Chino, CA, USA), stained with ethidium bromide and photographed (Unipro Platinum 2.0 Uvitec, Cambridge, UK).

**Table-I:** Primer sequences and amplicon sizes

*Genes*	*Primer Sequence (5' to 3')*	*Amplicon Size (bp)*	*References*
CTX-M	ATGTGCAGYACCAGTAARGTKATGGC	593	^10^
	TGGGTRAARTARGTSACCAGAAYCAGCGG		
TEM	ATGAGTATTCAACATTTCCG	867	^11^
	CTGACAGTTACCAATGCTTA		
SHV	CGCCGGGTTATTCTTATTTGTCGC	1015	^12^
	TCTTTCCGATGCCGCCGCCAGTCA		
OXA-1	ACACAATACATATCAACTTCGC	814	^12^
	AGTGTGTGTTTAGAATGGTGATC		
OXA-2	TTCAAGCCAAAGGCACGATAG	702	^12^
	TCCGAGTTGACTGCCGGGTTG		
OXA-10	CGTGCTTTGTAAAAGTAGCAG	651	^12^
	CATGATTTTGGT GGGAATGG		

***Statistical analysis: ***Fisher’s exact test was used as a statistical tool for comparisons of proportion between two groups of ESBLs producing isolates collected in two different time periods, respective to their source samples. All data were assessed using two tailed P value. 

## RESULTS

**Proportion of ESBL producing** ***E. coli*** **: **A statistically significant drift towards high prevalence of ESBLs production in *E. coli* isolates was observed from year 2005 to 2009-10 regardless of the sample sources of the isolates as the figure jumped from 34% in 2005 to 60% in 2009-10 (*P *= .0008). The proportion of ESBL producers among *E. coli *isolates collected from urine in 2009-10 was significantly higher (62.9%) as compared to those isolated in 2005 (31.8%) (*P *= .0009). However, the change was not remarkable in pus isolates; 41.1% in 2005 and 55.5% in 2009-10. Detailed results are shown in Table-II.

**Table-II:** Phenotypic confirmation of ESBL producing *E. coli* isolates

	*2005*		*2009-10*	
Specimen	*Tested Isolates*	*ESBL producers (%)*	*Tested Isolates*	*ESBL producers (%)*
Urine	66	21 (31.8)	54	34 (62.9)
Pus	17	7 (41.1)	36	20 (55.5)
Total	83	28 (33.7)	90	54 (60.0)

***Antimicrobial Susceptibility Testing: ***All ESBL producing *E. coli *isolates exhibited high level of resistance towards cefotaxime, ceftazidime, ciprofloxacin, gentamicin, nalidixic acid, ticarcillin-clavulanic acid and trimethoprim-sulfamethoxazole, but were highly susceptible to fosfomycin and imipenem. Antimicrobial resistance pattern was almost similar in all isolates from years 2005 and 2009-10 with minor differences except for fosfomycin, imipenem and colistin sulphate. Fosfomycin resistance was not detected in any of 2005 isolates but 5.8% urinary tract and 15% pus isolates were found resistant among 2009-10 isolates. Imipenem resistance was not detected in any of pus isolates from both batches, however, nearly 2.9% urinary tract isolates from 2009-10 showed resistance, whereas none was seen in 2005 isolates. Highly significant increase was observed in colistin sulphate resistance with respective figures of 9.5% and 64.7% for urinary isolates (*P *= .0001); and 0% and 55.0% for pus isolates (*P *= .0216). Interestingly some decrease in gentamicin resistance was observed but it was not statistically significant. Details are given in Table-III.

**Table-III:** Antimicrobial resistance of ESBL producing *E. coli*

*Antibiotic*	*Urine samples*		*Pus Samples*	
	*2005(n=21)* *R (%)	*2009-10(n=34)* *R (%)	*2005(n=7)* *R (%)	*2009-10(n=20)* *R (%)
Imipenem	0	2.9	0	0
Fosfomycin	0	5.8	0	15.0
Colistin Sulphate	9.5	64.7	0	55.0
Cefotaxime	95.2	100	100	100
Ceftazidime	95.2	100	85.7	100
Ciprofloxacin	90.4	100	100	95.0
Gentamicin	95.2	85.2	85.7	80.0
Nalidixic Acid	95.2	100	100	100
Ticarcillin Clavulanic Acid	100	94.1	100	90.5
Trimethoprim-Sulfamethoxazole	100	94.1	100	95.0

*R= resistance

**Molecular Characterization of ESBLs producing** ***E. coli: ***Detection level of ESBL related genes among all ESBL producing isolates increased significantly from 2005 to 2009-10, respective figures being 4% and 43% for *bla*_CTX-M_ (*P *= .0001); and 11% and 48% for *bla*_TEM _(*P *= .0007). Simultaneous occurrence of *bla*_CTX-M_ and *bla*_TEM_ genes was detected in 4% isolates from 2005 but the figure rose significantly to 30% (*P* = .0080) in isolates from 2009-10. The targeted SHV and OXA gene fragments were not found in any of the isolates.

No CTX-M or TEM gene was detected in *E. coli *isolates from pus samples collected in year 2005 whereas in 2009-10 each of these genes was detected in 35.0% *E. coli* isolates whereas in 20% isolates, both of these genes were detected. Similarly very low prevalence of CTX-M and TEM was observed in isolates from urine collected in 2005; 4.7% and 14.2%, respectively, whereas both genes were present simultaneously in 4.7% of the isolates. Among pathogens isolated from urine in 2009-10, 47.0% (*P *= .0009) were conferring resistance due to CTX-M genes whereas TEM gene was detected in 55.8% (*P *=.0040) of the ESBL producing isolates. Both CTX-M and TEM were simultaneously detected in 35.2% (*P *=.0102) of the isolates. Detailed results are shown in Table-IV.

**Table-IV:** Distribution of genes encoding CTX-M and TEM enzymes

	*2005*				*2009-10*			
*Specimen*	*Isolates (n)*	*CTX-M (%)*	*TEM (%)*	*CTX-M & TEM (%)*	*Isolates (n)*	*CTX-M (%)*	*TEM (%)*	*CTX-M & TEM (%)*
Urine	21	1(4.7)	3(14.2)	1(4.7)	34	16(47.0)	19(55.8)	12 (35.2)
Pus	7	0	0	0	20	7(35.0)	7(35.0)	4 (20.0)
Total	28	1(3.5)	3(10.7)	1(3.5)	54	23(42.5)	26(48.1)	16 (29.6)

## DISCUSSION

In this study, a rapid increase in incidence of ESBL producing *E. coli* was noted from year 2005 (33.7%) to 2009-10 (60.0%) **(**Table-II**)**. Although the figures for 2005 are comparable with other reports of that period, e.g., 32% from Japan^13^ and 31.9% from Italy,^14^ the respective figures (60.0%) for 2009-10 were much higher than contemporary reports from Eastern Europe (10%), Western Europe (6%), Latin America (6%), Brazil (17.3%),^15^ North America (3%), India (32%), Tanzania (24.4%)^16^ and Asia Pacific (8.6%).^17^ It is also noticeable that these figures were in fact on decline in the above mentioned countries but in Pakistan the increase from 2005 to 2009-10 was nearly twofold. It is an alarming situation and when we consider the development of resistance against commonly used drugs, the situation is very pessimistic. Only encouraging observation was that the resistance towards gentamicin decreased slightly from more than 90% to nearly 80%.

The situation is relatively better when we look at the performance of drugs especially used against ESBL producing bacteria. With the exception of colistin sulphate which registered a steep increase in resistance from 2005 to 2009-10 (from less than 10% to more than 50%), imipenem and fosfomycin were still very effective (Table-III). However, the point for worry is that there were no resistant isolates in 2005 but now these are emerging conspicuously. The current level of imipenem resistance is comparable to recent global reports,^7^^,^^16^ but the rise in colistin sulphate resistance is remarkably higher than that reported in contemporary studies.^18^ Similarly, rise in fosfomycin resistance is greater as compared with recent reports.^19^

When we compare the urinary isolates with pus isolates, no significant difference was seen in pattern shift from 2005 to 2009-10 (Table-III). Only notable points were that imipenem resistance was still absent from pus isolates whereas fosfomycin resistance was more visible in pus isolates.

As is well known, the number of genes encoding ESBLs is large,^20^ and it is impossible to cover them all in one study. In this study, CTX-M type ESBLs were detected in 43% of the ESBL producing *E. coli* isolates. These figures are slightly lower than the figure of 59% reported in a recent study.^7^ In contrast, the prevalence of TEM encoding genes was 48% in ESBL producing isolates in this study. This figure is much higher than a recent report from Pakistan which showed figures of only 21%.^7^ Occurrence of the isolates carrying both *bla*_TEM_ and *bla*_CTX-M_ genes simultaneously (47.2%) was comparable to a recent study in Brazil.^15^ Our striking finding was the rapid rise of ESBL producing *E. coli* harboring CTX-M and TEM genes. The respective figures for CTX-M, TEM, and CTX-M + TEM were 4%, 11% and 4% in 2005; and 43%, 48% and 40% in 2009-10. The rising trend has been reported in other comparative studies as well.^1^^,^^4^

The characteristics of pathogens in different parts of the world cannot be similar. These characteristics depend on social practices, hygienic conditions and access of common man to antimicrobials. In most of developing countries, these drugs are freely available and unnecessary usage is common. Other factor is that due to poverty, treatment is abundant as soon as some recovery is noticed. This is a major factor in rapid development of drug resistance. 

Our results show that ESBL producing *E. coli* isolated from urinary and wound infections from this region are very versatile. These were already highly resistant to conventional drugs including, cefotaxime, ceftazidime, ciprofloxacin, gentamicin, nalidixic acid ticarcillin clavulanic acid and trimethoprim-sulfamethoxazole in 2005, but showed no or very little resistance to imipenem, fosfomycin and colistin sulphate. During five years, rapidly emerging resistance against these drugs was apparent, especially colistin sulphate. It was also noted that the acquisition of this resistance was much rapid than other parts of the world. Our study underlines the importance that should be given to the emerging threat of ESBL producing *E. coli* not only with reference to local situation but global scenario as well because in these days of extensive traveling and keeping in view the versatility of these pathogens, with horizontal transfer as a main tool, these traits can spread not only to *E. coli* isolates in other regions but also to other bacterial pathogens.

## Authors Contribution:

Muhammad Asif Habeeb: Main laboratory work, collection of samples, manuscript writing.

Yasra Sarwar: Guidance in laboratory work related to microbiology.

Aamir Ali: Guidance in laboratory work related to molecular biology.

Muhammad Salman: Sample collection and help in laboratory work.

Abdul Haque: Concept, design of work and finalizing the manuscript.
